# 139. Clinical Predictors of a Positive MPox PCR: A Framework for Diagnostic Stewardship

**DOI:** 10.1093/ofid/ofad500.212

**Published:** 2023-11-27

**Authors:** Adrienne M Gonzales, Rose Yeh, ikram Rostane, Hunter Ratliff, Audrey Wanger, Luis Ostrosky-Zeichner

**Affiliations:** University of Texas Health Science Center- Houston, Houston, Texas; University of Texas Health Science Center- Houston, Houston, Texas; University of Texas Health Science Center- Houston, Houston, Texas; University of Texas Health Science Center- Houston, Houston, Texas; McGovern Medical School, Houston, Texas; McGovern Medical School. UTHealth, Texas, Texas

## Abstract

**Background:**

In July 2022, an outbreak of MPox was reported in the United States as part of a world-wide health emergency. Healthcare settings in the US started to see possible cases, and the public health and clinical diagnostics infrastructure was quickly overwhelmed. As the concept of diagnostic stewardship is becoming increasingly more important, we sought to identify clinical information obtainable at presentation that would predict a positive MPox PCR in order to streamline testing and optimize resource utilization.

Table 1

**Figure:**
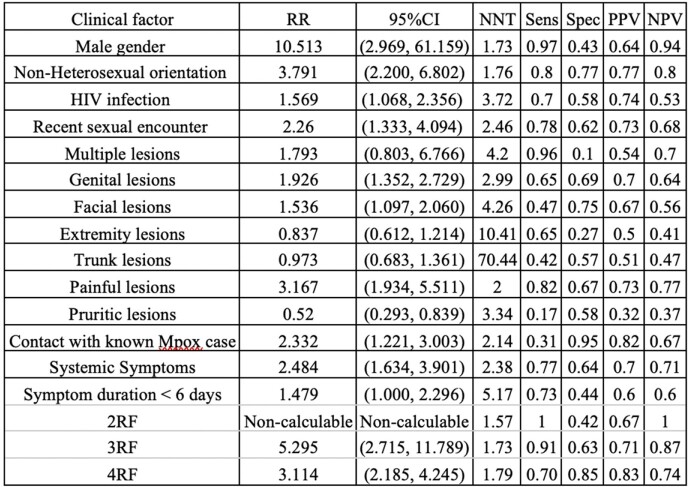

RR and diagnostic performance for single and combined clinical factors resulting in a positive MPox PCR

**Methods:**

The charts of 146 patients presenting for MPox testing in a large healthcare system in Texas in July and August 2022 were reviewed to identify clinical factors upon presentation that would correlate with a positive lesion PCR.

**Results:**

49.3% of patients tested positive. The mean (SD) age of patients was 31.9 (8.41) years. The mean (SD) time of symptoms was 5.47 (5.08) days. The mean (SD) time from last sexual encounter was 17.6 (11.01) days. Table 1 shows the RR and diagnostic performance for each clinical factor resulting in a positive MPox PCR as well as the RR for those with 2, 3, and 4 combined high risk clinical factors.

**Conclusion:**

Male gender, non-heterosexual sexual orientation, recent sexual encounter, painful lesions, contact with a known MPox case, systemic symptoms, and shorter symptom duration less than or equal to 6 days increased the risk of a positive Mpox PCR result and may be used to prioritize testing in a resource constrained environment. Patients presenting with a combination of 2, 3, and 4 risk factors further increased the likelihood of a positive test. Risk factors can be used for diagnostic stewardship in MPox testing. Further research will focus on creation and prospective validation of clinical prediction rules.

**Disclosures:**

**Luis Ostrosky-Zeichner, MD, FACP, FIDSA, FSHEA, FECMM, CMQ**, Astellas: Grant/Research Support|Cidara: Advisor/Consultant|Cidara: Advisor/Consultant|F2G: Advisor/Consultant|Gilead: Advisor/Consultant|Gilead: Grant/Research Support|GSK: Advisor/Consultant|Melinta: Advisor/Consultant|NIH: Grant/Research Support|Pfizer: Advisor/Consultant|Pfizer: Grant/Research Support|Pfizer: Honoraria|Pulmocide: Grant/Research Support|Scynexis: Grant/Research Support|T2 Biosystems: Grant/Research Support|Viracor: Advisor/Consultant

